# Effects of a 16-Week Digital Intervention on Sports Nutrition Knowledge and Behavior in Female Endurance Athletes with Risk of Relative Energy Deficiency in Sport (REDs)

**DOI:** 10.3390/nu15051082

**Published:** 2023-02-21

**Authors:** Ida L. Fahrenholtz, Anna K. Melin, Ina Garthe, Siri Marte Hollekim-Strand, Andreas Ivarsson, Karsten Koehler, Danielle Logue, Petra Lundström, Sharon Madigan, Paulina Wasserfurth, Monica K. Torstveit

**Affiliations:** 1 Department of Sport Science and Physical Education, University of Agder, 4630 Kristiansand, Norway; 2 Department of Sport Science, Linnaeus University, 35252 Växjö, Sweden; 3 The Norwegian Olympic and Paralympic Committee and Confederation of Sport, 0854 Oslo, Norway; 4 Department of Neuromedicine and Movement Science, Norwegian University of Science and Technology, 7034 Trondheim, Norway; 5 School of Health and Welfare, Halmstad University, 30118 Halmstad, Sweden; 6 Department of Sport and Health Sciences, Technical University of Munich, 80809 Munich, Germany; 7 Sport Ireland Institute, Sport Ireland Campus, Abbotstown, D15 PNON Dublin, Ireland; 8 Department of Health Science, Karlstad University, 65188 Karlstad, Sweden; 9 Department of Molecular Medicine and Surgery, Karolinska Institute, 17176 Stockholm, Sweden

**Keywords:** athletic injuries, digestion, female athlete triad, diet therapy, endurance training, menstruation disturbances, women’s health

## Abstract

Female endurance athletes are considered a high-risk group for developing Relative Energy Deficiency in Sport (REDs). Due to the lack of educational and behavioral intervention studies, targeting and evaluating the effects of the practical daily management of REDs, we developed the Food and nUtrition for Endurance athletes—a Learning (FUEL) program, consisting of 16 weekly online lectures and individual athlete-centered nutrition counseling every other week. We recruited female endurance athletes from Norway (*n* = 60), Sweden (*n* = 84), Ireland (*n* = 17), and Germany (*n* = 47). Fifty athletes with symptoms of REDs and with low risk of eating disorders, with no use of hormonal contraceptives and no chronic diseases, were allocated to either the FUEL intervention (*n* = 32) (FUEL) or a 16-week control period (*n* = 18) (CON). All but one completed FUEL, while 15 completed CON. We found strong evidence for improvements in sports nutrition knowledge, assessed via interviews, and moderate to strong evidence in the ratings concerning self-perceived sports nutrition knowledge in FUEL versus CON. Analyses of the seven-day prospective weighed food record and questions related to sports nutrition habits, suggested weak evidence for improvements in FUEL versus CON. The FUEL intervention improved sports nutrition knowledge and suggested weak evidence for improved sports nutrition behavior in female endurance athletes with symptoms of REDs.

## 1. Introduction

Female endurance athletes are at high risk of low energy availability (LEA) and related consequences, including menstrual dysfunction, gastrointestinal problems, and injuries [1,2]. The multiple health- and performance-related consequences of LEA are collectively termed Relative Energy Deficiency in Sport (RED-s [2,3] with the female athlete triad historically laying the foundation for the RED-S model [4].

There are many reasons for LEA ranging from disordered eating and eating disorders to unintentional under fueling [5]. One of the most prevalent underlying causes of LEA- and RED-S-related symptoms has been reported to be unintentional [6,7], due to the suppression of appetite after high- and moderate-intensity training [8,9] and/or a lack of knowledge of optimal sports nutrition, and the consequences of LEA over time [10,11,12,13,14]. In addition, there seems to be a normalization of some of the most frequent RED-S conditions, preventing female athletes from seeking help, for e.g., menstrual dysfunction [15].

Dietary characteristics associated with LEA and menstrual dysfunction include a high dietary fiber intake [16,17,18], a low fat intake [16,18,19], and a low intake of carbohydrates [16]. While dietary fiber and protein intake have been reported to exceed general and sports nutrition recommendations among female endurance athletes [16], the carbohydrate intake is generally below sports nutrition recommendations [16,20,21]. Though several factors influence food choices in athletes [22,23], nutrition knowledge is one of the few determinants of dietary behavior that are modifiable and, therefore has the potential to impact athletic health and performance [23]. More specifically, sports nutrition knowledge has been positively associated with energy availability and carbohydrate intake in female endurance athletes [24], and educational initiatives have been proposed as a promising strategy to prevent and improve symptoms of RED-S [10,25].

Clinical symptoms of RED-S are concerning, with potential significant impacts on the individual’s quality of life and requiring a highly resource demanding treatment. Therefore, early prevention and management of RED-S in female athletes is of key interest [26], due to the high prevalence, ranging from 22–58% in various sports [11], and the potential irreversible health consequences, such as osteopenia and premature osteoporosis [27,28]. Given that sports nutrition knowledge has been reported to be inadequate in endurance athletes [29], improving sports nutrition knowledge could represent an important step in the management of RED-S.

Planning and delivering a sports nutrition intervention in an athlete-based population is complex, when you compare it with developing and producing practical guidelines for athletes in endurance sports with already established routines and a rigid seasonal competition plan. In addition, there are other factors to consider, such as individual taste preferences, social influences, cultural background, time, cost–benefit evaluations related to financial status, and psychological factors [22,30,31]. All these factors can influence athletes’ food choices and dietary behavior, emphasizing the importance of an individual approach, and that the intervention should not only aim for improved nutrition knowledge, but also to provide the basis for skill and competence improvements, by motivating, enabling and supporting athletes, making them able to implement the acquired knowledge in their everyday lives [25,30].

The mode of delivery is another significant factor to consider when initiating interventions for athletes. Digital interventions may offer advantages due to for e.g., money and time saving from transportation, and lower attrition rates [32].

The recommended management of athletes with RED-S symptoms is to ensure adequate energy intake relative to energy expenditure [33,34,35], although evidence on the efficacy of this approach is limited and primarily based on case studies [36,37,38], interventions without a control group [39,40], and in non-athletic populations [41].

Practice-orientated low-cost RED-S management strategies with sustainable potential would, therefore, be attractive for a future implementation in real-life settings. To progress such efforts, first, programs need to be developed and tested. Focusing on high-risk groups, namely female endurance athletes with symptoms of RED-S, appears to be a good starting point for investigating such strategies.

Therefore, the aim of the present international multicenter study was to develop, implement and evaluate a 16-week nutrition intervention, consisting of online sports nutrition lectures combined with individual nutrition counseling for female endurance athletes at risk of RED-S. Specifically, the goal of this analysis was to investigate whether the sports nutrition knowledge and dietary outcomes would change differently from the baseline to post-intervention in the intervention group compared to the control group.

## 2. Materials and Methods

It has been proposed that educational initiatives targeting athletes with RED-S should underscore the positive aspects of energy, namely, that food is *fuel* and *fuel* is needed for performance [25]. Therefore, we called the project Food and nUtrition for Endurance athletes—a Learning program (the FUEL project). The study was registered at www.clinicaltrials.gov (NCT04959565) and was approved by the regional ethics committee in Norway (31640), Sweden (2019-04809), and by the Norwegian Centre for Research Data (968634). Originally, the study was planned and approved to include a wide range of RED-S related clinical biomarker measurements and a control group prior to initiation of the intervention. Due to the COVID-19 pandemic all physical contact with the participants was prohibited and the final design and methods are strongly influenced by the pandemic restrictions. Since the final research plan included no medical procedures, the study was considered exempt from additional ethical approval at the other study sites (Germany and Ireland).

### 2.1. Study Design

The FUEL project was a non-randomized multicenter study including female endurance athletes from Norway, Sweden, Ireland, and Germany. The FUEL intervention group received weekly lectures in sports nutrition combined with fortnightly individual athlete-centered nutrition counseling with an experienced sports nutritionist, while the control group received no lectures or counseling. Because many female endurance athletes at both the regional and national level know each other, the risk of imitation of the intervention was considered to be high. Therefore, seasonal allocation of summer and winter sport disciplines was prioritized over randomization, and the athletes from summer and winter sport disciplines, respectively, received the intervention simultaneously.

**Figure 1:**
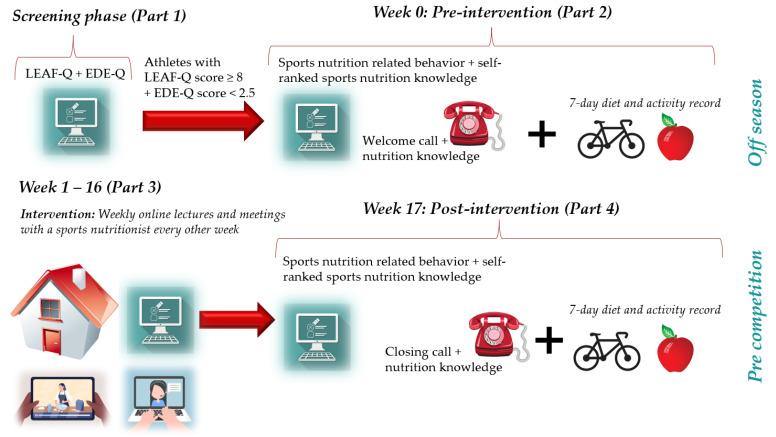
Overview of the study design with measures relevant to this analysis. Athletes who volunteered were asked to complete the Low Energy Availability Questionnaire (LEAF-Q), the Eating Disorder Examination Questionnaire (EDE-Q), and background information (Part 1). Athletes with a LEAF-Q score ≥ 8 and an EDE-Q global score < 2.5 were invited to further participation and asked to record food intake and physical activity including training for seven consecutive days, and answer questions related to sports nutrition behavior and self-perceived sports nutrition knowledge. At the beginning of the pre-intervention week, athletes received a welcome call where they were asked to reply to statements for the assessment of sports nutrition knowledge (Part 2). A 16-week intervention was conducted (Part 3) with weekly sports nutrition lectures in combination with athlete-centered nutrition counseling with an experienced sports nutritionist every other week followed by post-intervention data collection (Part 4). The control group went through the same pre- and post-assessments without any lectures or nutrition counseling. Part elements from Colourbox.

The study was initiated with a screening phase (Figure 1), where athletes completed an online survey (Part 1) via the safe data collection tool Nettskjema that was connected to the Services for Sensitive Data (TSD) platform (University of Oslo), which collected data including background information, training volume (average hours/month), and included the LEA in Females Questionnaire (LEAF-Q) [1] and the Eating Disorder Examination Questionnaire (EDE-Q) [42]. In this group of athletes, the Cronbach’s alpha coefficients ranged from 0.51 (menstrual function) to 0.68 (injuries) on the LEAF-Q subscales, and from 0.82 to 0.94 on the EDE-Q subscales. Detailed information regarding Part 1 of the study has previously been published [7]. Athletes with a risk of LEA (LEAF-Q score ≥ 8) [1] and a low risk of disordered eating behavior (EDE-Q global score < 2.5) [43,44] were invited to complete an additional survey, including questions regarding sports nutrition-related behavior and self-perceived sports nutrition knowledge. In the same week that a seven-day dietary and training record (Part 2) was conducted, a telephone interview with questions regarding sports nutrition knowledge was performed. Athletes who signed up during their competition season and fulfilled inclusion criteria, were allocated to a waiting list with control group conditions, before they were offered the intervention with/without individual counseling (data from the control group’s intervention are not included in the present analyses).

Study week 0 (baseline) was followed by the intervention (Part 3), during the athletes’ off-season or a 16-week control period (study week 1–16). The duration from Part 1 to Part 2 was approximately four weeks. After the 16-week intervention or control period, athletes once again completed the two online surveys, the telephone interview, and a seven-day diet and activity record (Part 4, week 17).

### 2.2. Inclusion and Exclusion Criteria

Participants were non-smoking, female endurance athletes, 18–35 years of age, trained a minimum of five times per week, tier 3–4 [45], and competed in one of the following endurance disciplines: long-distance running, orienteering, cycling, triathlon, cross-country skiing, or biathlon. The exclusion criteria were: the use of hormonal contraceptives, chronic diseases (e.g., Crohn’s disease or hypothyroidism), pregnancy, and menstrual dysfunctions not related to LEA. Athletes using hormonal contraceptives were included if they discontinued the use at least six weeks before the screening phase (Part 1).

### 2.3. Recruitment and Eligibility

Athletes were recruited from November 2020 to September 2021 via Norwegian, Swedish, Irish, and German competitive endurance sports clubs, namely the Norwegian Olympic Sport Centre, the Sport Ireland Institute, the Swedish National Sport Federation, Swedish sport federations within endurance sports, the German Ski Federation, and the German Olympic Sports Confederation, and via social media with a link to the project website and the Part 1 survey [7]. The recruitment targeted summer endurance disciplines (runners, orienteers, cyclists, and triathletes) during November/December with the initiation to the intervention in January, while the recruitment targeted winter endurance disciplines (biathletes and cross-country skiers) in May with the initiation to the intervention in June.

**Figure 2:**
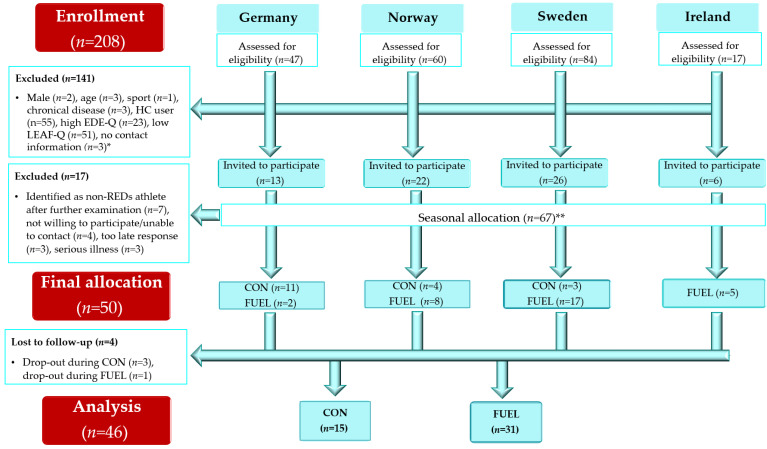
Flow chart. * Several participants had more than one reason for exclusion, e.g., hormonal contraceptives and a high EDE-Q global score. The primary cause for exclusion is based on the given order. ** Athletes were allocated to the intervention in their off-season. Athletes who signed up during their competition season were allocated to a waiting list control group condition. Abbreviations: CON: athletes participating in the 16-week control period; EDE-Q: Eating Disorder Examination Questionnaire; FUEL: Food and nUtrition for Endurance athletes—a Learning program; HC: hormonal contraceptives; LEAF-Q: Low Energy in Females Questionnaire; RED-S: Relative Energy Deficiency in Sport.

In total, 208 participants completed Part 1 of the study and were assessed for eligibility for further participation (Figure 2). One hundred and forty-one athletes were excluded: *n* = 2 male athletes; *n* = 2 < 18 years; *n* = 1 > 35 years.; *n* = 1 badminton player; *n* = 3 with chronic diseases [*n* = 1: Crohn’s disease, *n* = 1: Hashimoto’s thyroiditis, *n* = 1: hypothyroidism]; *n* = 55 hormonal contraceptive users; *n* = 23 with a EDE-Q global score ≥ 2.5; *n* = 51 with a LEAF-Q score < 8, and *n* = 3 for not providing any contact information. All excluded participants with available contact information (*n* = 138) were contacted by e-mail and given the opportunity to receive the reason for their exclusion via a telephone call from the researchers or in an encrypted file sent by email. All participants with EDE-Q ≥ 2.5 (*n* = 43) were informed and encouraged to contact their general practitioner for further examination, and were provided with links to relevant web pages, including voluntary associations that offer help to people with disordered eating behavior or eating disorders.

The LEAF-Q responses of *n* = 67 athletes were analyzed in more detail, and some were contacted to clarify their answers. This resulted in *n* = 7 athletes being excluded due to a suspected false positive identification of the risk of RED-S. Further, *n* = 4 athletes were unavailable, *n* = 3 responded too late in relation to intervention start-up and allocation to sports nutritionists, and *n* = 3 athletes declared severe illness ahead of the baseline measurements (e.g., abdominal surgery and COVID-19). In total, *n* = 18 athletes were allocated to a 16-week waiting list control condition of which *n* = 15 athletes completed (*n* = 1 wanted to start using hormonal contraceptives, while we were unable to make contact with *n* = 2). In total, *n* = 32 athletes were directly allocated to the FUEL intervention, while *n* = 1 terminated participation in the project in week 13 due to experiencing too much work related to the project. Consequently, *n* = 31 (97%) completed the FUEL intervention and *n* = 15 (83%) completed the control condition.

### 2.4. Nutrition Intervention

**Figure 3:**
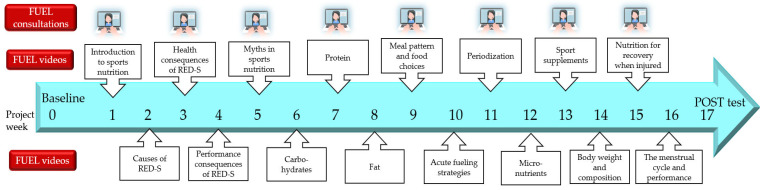
Overview of the FUEL nutrition intervention with weekly lectures in sports nutrition (FUEL videos) and athlete-centered nutrition counseling (FUEL consultations) every other week. Part elements from Colourbox.

The 16-week intervention consisted of weekly online lectures in sports nutrition targeting female endurance athletes with a risk of RED-S, combined with individual athlete-centered nutrition counseling every other week. Figure 3 represents an overview of the FUEL intervention.

#### 2.4.1. Sports Nutrition Lectures

The sixteen sports nutrition lectures integrated evidence-based sports nutrition information and recommendations [2,46,47,48] (Figure 3). The lectures were developed by four researchers and practicing sports nutritionists, initially in Norwegian and Swedish, including a comprehensive manuscript for each session, and subsequently translated into English and German. All sixteen lectures were comprehensively reviewed and finally approved by all four researchers. The lectures were recorded by experienced female sports nutrition researchers and averaged 25.0 ± 8.4 min in duration (range: 15–43 min, total duration of 400 min). Every week during the intervention, participants received an email with a link and password to the lecture of the week located on a closed online platform. Participants had the opportunity to watch the lectures when suitable during their everyday lives and to watch them repeatedly if they wanted. In the development of the lectures, emphasis was placed on the dissemination of: sports nutrition research in easily understandable language, scientific sports nutrition recommendations with practical examples, relevant and explanatory pictures, case stories, as well as the benefits of optimal nutrition for health and performance, and the potential consequences of inadequate fueling. The lectures enabled behavior change techniques, such as “4.1 instruction on how to perform the behavior”, “4.2 information about antecedents”, “5.1 information about health consequences”, “5.2 salience of consequences”, and “5.5. anticipated regret”, according to the behavior change technique taxonomy (v1) defined by Michie et al. [49].

The first lecture included a general introduction to sports nutrition for endurance athletes (Figure 3). The following three lectures covered the potential underlying causes, the health-related, and performance-related consequences of RED-S, respectively. The aim of the fifth lecture was to address typical myths in the field of sports nutrition, followed by lectures on macronutrients, meal patterns, and food choices. The tenth lecture (nutrition for performance) covered acute fueling strategies, i.e., before, during, and after training and competition, followed by lectures about periodization, micronutrients, and sport nutrition supplements. The fourteenth lecture included a discussion on the impact of body weight and body composition on health and performance, while the fifteenth lecture presented optimal nutrition strategies to implement when injured. The intervention was completed with a lecture focused on the importance of regular menstruation, and the potential effects of the different menstrual cycle phases on athletic performance.

#### 2.4.2. Athlete-Centered Nutrition Counseling

The nutrition counseling was administrated via the teleconferencing platform Zoom, Zoom Video Communication, Inc. (San Jose, CA, USA). The first consultation was scheduled to run for 1.5 h, while the following seven consultations were scheduled to run for approximately 1 h. The actual mean duration for the first consultation was 73 ± 15 min, and 55 ± 6 min for the remaining consultations.

The FUEL counseling team consisted of three Norwegian, four Swedish, two Irish, and one German highly experienced sports nutritionists. To improve standardization, a comprehensive manual was developed, and three webinars were conducted ahead of the intervention, in addition to weekly Zoom meetings by the FUEL counsellor team during the course of the intervention. The weekly meetings were guided by the head of nutrition at the Norwegian Olympic and Paralympic Committee and the Confederation of Sports, and the overall data collection coordinator. Because a self-determination theory approach has been found to be effective in promoting behavior change through internal sources of motivation [50], autonomy in particular, but also competence and relatedness, were a core foundation in the FUEL counseling. To achieve this, a client-based, empathic communication approach, inspired by core skills in motivational interviewing, was utilized [51]. Athletes were introduced to the transtheoretical model of behavior change [52] in lecture 1. During the first consultation with the athlete, the sports nutritionist strived to raise the athlete’s awareness of readiness to change through empathic, athlete-centered dialogue. If applicable, the athlete was asked to define her readiness for change, depending on the behavior(s) in question, by placing herself in the transtheoretical model of behavior change. The consultations were customized from one consultation to the next depending on the individual athlete’s readiness for change.

**Figure 4:**
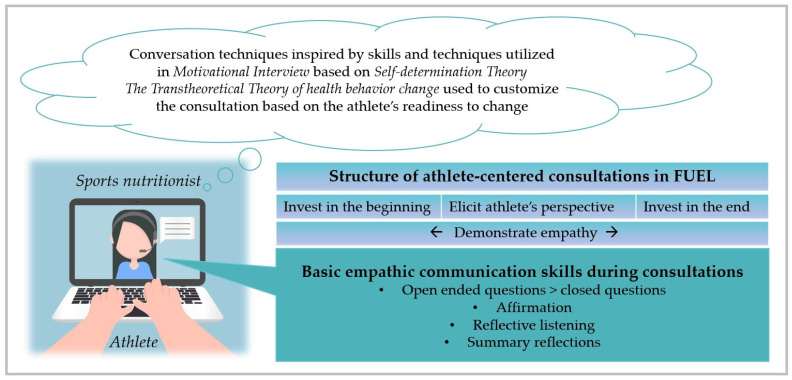
How to promote athlete-centered communication in FUEL based on [51,52,53]. Part element from Colourbox.

An illustration of how to promote athlete-centered communication is presented in Figure 4. The structure of the FUEL consultations was inspired by the Four Habits Model [53,54] and is presented in Table 1.

The FUEL nutrition counselling manual included a description of the theoretical foundation of FUEL, the counseling approaches customized according to the perceived readiness for change, a framework for the eight consultations, examples of ambivalence exploration, and how to give individual athlete-targeted information. Furthermore, a decision flow diagram, based on the RED-S CAT [55], concerning when athletes should be encouraged to seek an examination outside the FUEL project (e.g., a gynecologist relating to an undiagnosed menstrual dysfunction) was included. An experienced psychiatrist, specializing in eating disorders in both athletes and non-athletic populations, was affiliated as the medical professional responsible within the project, supporting the FUEL counselors if needed. Athletes were encouraged to write reflection notes between the sessions. In addition, counselors were provided with the athlete’s LEAF-Q response and a table to fill in the athlete’s self-formulated nutrition goals. The date, time, and actual duration of the session were noted for all eight sessions, in addition to the athlete’s goal for the next session, and any additional comments from the session in question. Counselors had access to the online lectures and a printout of the PowerPoint presentations with the manuscript, in order for them to prepare for any questions related to the latest topics in the FUEL lectures.

Because the consultations were customized according to the individual athlete’s needs and preferences, different behavior change techniques were enabled and were, among others, designed to include, “1.1 goal setting (behavior)”, “1.2 problem solving”, “1.5 review behavior goal(s)”, “1.9 commitment”, “2.2 feedback on behavior”, and “3.3 social support (emotional)”, according to the behavior change technique taxonomy (v1) defined by Michie et al. [49].

### 2.5. Sports Nutrition Knowledge

Although questionnaires to assess nutrition knowledge have been validated among endurance athletes [56], this method of measurement was considered inappropriate for this study, where physical attendance was not possible and thus, researchers could not control whether participants searched for the correct answers in books or online. Instead, twenty statements that were suitable to be read out during a telephone interview were developed with the possibility of answering “true”, “false”, or “unsure”. The number of correct answers was assessed at the baseline and post-intervention. In addition, participants were asked to rank their sports nutrition knowledge on five statements in an online survey on a scale from 1 to 10 (1 = totally disagree, 10 = fully agree). The twenty statements used in the telephone interview and the five questions concerning self-ranked sports nutrition knowledge were pilot tested with a small group of female endurance athletes in terms of relevance and readability before the initiation of the study.

### 2.6. Sports Nutrition-Related Behavior and Dietary Intake

Based on current sports nutrition recommendations [46,47], ten questions related to sports nutrition behavior were developed. To enable an assessment of any developments from the baseline to the post-intervention/control period, a scoring system (see Supplementary File S1) was developed. The total score ranged from 0–30, where 30 indicated the best fulfillment of the sports nutrition recommendations. As some of the questions could be regarded as irrelevant for some athletes, a “not relevant” box was applied for two of the questions, and the final score was then divided by the answers available, making 3.0 the highest possible global score. The scoring system was developed in the context of what is considered the optimal approach for athletes with symptoms of LEA.

Dietary intake was calculated from a seven-day weighed food record. Participants received a kitchen scale via the postal service, including a user’s manual with instructions and a demonstration with pictures on how food and drink should be weighed and recorded. In addition, all participants received a telephone call to ensure that they understood how to conduct the dietary registration correctly. All completed dietary records were reviewed by one of the project members who asked the athletes for in-depth answers when needed, for e.g., when under- or overreporting was suspected. The Norwegian and Swedish participants registered all food and beverages in the Dietist Net Matdagbok (Kost och Näringsdata AB, Bromma, Sweden), in which the participants were unable to see any nutritional content when registering the food and beverages. The data were subsequently analyzed using Dietist Net Pro [57]. The Irish and German participants registered food and beverages in paper form before a researcher entered the data into Nutritics (2019, Research Edition v5.09, Dublin, Germany) and EBISpro [58] (2016, University of Hohenheim, Stuttgart, Germany) [59], respectively.

For each day, carbohydrate intake (g/kg body mass) was compared to current carbohydrate recommendations for endurance athletes [46,47,60,61]. Thus, the daily carbohydrate intake was assessed relative to the training volume of the day. The following criteria for meeting carbohydrate intake recommendations were used: training < 0 h/day: minimum 4 g/kg; training 0.5–1.5 h/day: minimum 6 g/kg; training 1.6–3.9 h/day: min. 7 g/kg; training ≥ 4 h/day: min. 9 g/kg.

### 2.7. Physical Activity and Training

Participants were instructed to use a chest-worn heart rate monitor during all training sessions and to register all training in the Bestr online training diary (www.bestr.no), in as much detail as possible during the same seven days as the weighed food record. Athletes, who within the latest year, had performed a maximal heart rate test in a laboratory setting (*n* = 13), entered their maximal heart rate (HR_max_) manually into Bestr, whereas the remaining participants had their HR_max_ estimated in Bestr via the equation HR_max_ = 208 − 0.7 × age. The time in five intensity zones was calculated in Bestr: I1: 60–72% of HR_max_; I2: 60–72% of HR_max_; 72–82% of HR_max_; I3: 82–87% of HR_max_; I4: 87–92% of HR_max_; I5: 92–97% of HR_max_. Actigraphy (ActiGraph GT3X^®^, Pensacola, FL, USA) and the data analysis software ActiLife 5 (ActiGraph) were used for the assessment of non-exercise activity thermogenesis. Subjects were instructed to wear an accelerometer on their hip from getting up in the morning until bedtime, and only take it off during showering and training.

### 2.8. Statistics

Data analyses were conducted using JASP (version 0.16.3.0). All analyses were conducted within the Bayesian statistical framework [62]. In comparison to classical statistics, Bayesian statistics is less sensitive to multiple testing, reducing the risk of type I errors, and is also less sensitive to small sample sizes [62,63].

Descriptive statistics were expressed as frequencies with percentages for binary and categorical data, and as means ± standard deviation (SD) for continuous data. Group comparisons of the baseline characteristics were conducted using the Bayesian independent samples *t*-test for normally distributed data, and the Mann–Whitney test for non-normally distributed data. Group comparisons from week 0 to week 17 were conducted using Bayesian mixed factor analysis of variance (ANOVA) with default priors and compared to the null model. A group x time interaction effect was hypothesized, i.e., that the two groups’ nutrition knowledge would change differently over time (alternative hypothesis). To calculate the Bayes factor (BF) for the interaction effect, only inclusion probabilities for matched models were considered [64]. Bayes factors between 1 and 3 were considered to indicate weak evidence of the alternative hypothesis, BFs between 3 and 10 were considered moderate evidence of the alternative hypothesis, while BFs greater than 10 were considered strong evidence of the alternative hypothesis [65].

## 3. Results

The mean age of the included athletes was 24.9 ± 4.7 years, with an average training volume of 47.1 ± 17.0 h per month (Table 2). The majority of the athletes (82%) were studying or working alongside their endurance sport participation, while the remaining 18% reported being full-time athletes. Thirty-three percent had attended secondary school as their highest level of education, 39% had attended university/college for less than 4 years, while 28% had attended university/college for 4 years or more. Seventy percent competed at club level, 18% were at the national team level, 8% were professionals, while 4% classified themselves as “others” (e.g., competing in an endurance sport but not affiliated with a club). Consequently, the majority of the participants were classified as Tier 3 athletes [45]. There was no evidence of statistical differences when comparing the two groups’ baseline characteristics (BFs < 1).

### 3.1. Sports Nutrition Knowledge

**Figure 5:**
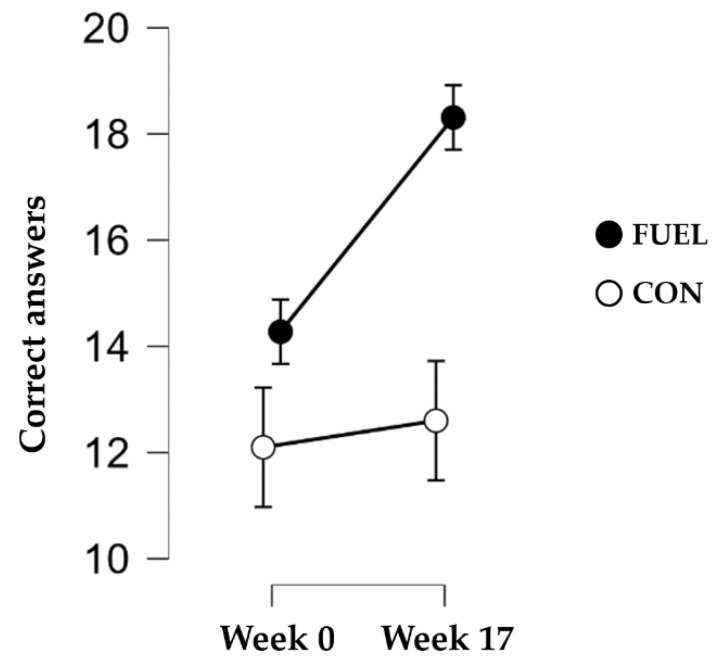
Correct answers from the telephone interview on sports nutrition knowledge. Data are presented as mean and 95% credible intervals. Abbreviations: CON: control group; FUEL: FUEL intervention group.

Figure 5 illustrates the number of correct answers from the telephone interview. Each statement and the distribution of correct answers can be found in Table S1. We found strong evidence of the alternative hypothesis, i.e., an interaction effect between the groups and measurement time point was present (BF_incl_ = 216.93).

Similarly, we found moderate to strong support for the alternative hypothesis, indicating an interaction effect between group and measurement time point, for four of the five questions related to self-perceived sports nutrition knowledge (Table 3).

### 3.2. Sports Nutrition-Related Behavior, Overall Dietary Intake, and Physical Activity

For the sports nutrition global score, we found weak evidence (BF_incl_ = 2.75) for the interaction effect between group and measurement time point (FUEL: 1.7 ± 0.5 at week 0 and 2.2 ± 0.4 at week 17; CON: 1.9 ± 0.4 at week 0 and 2.0 ± 0.4 at week 17). Similarly, we found weak support for an interaction effect between group and measurement time point for total energy intake, and carbohydrates (g/day and g/kg/day), protein (g/day and g/kg/day), and fat (E%) intake (Table 4).

Among athletes in the FUEL intervention group, 74% increased their carbohydrate intake compared to 44% in the control group (BF_incl_ = 1.266), while 61% in the FUEL intervention group increased their total energy intake compared to 56% in the control group (BF_incl_ = 0.341).

When each participant’s carbohydrate intake was compared to the current guidelines, the FUEL intervention group met the carbohydrate recommendations 1.2 ± 1.1 days/week at week 0 and 2.6 ± 1.8 days/week at week 17, while the control group met the carbohydrate recommendations 1.4 ± 1.2 at week 0 and 1.8 ± 1.8 at week 17 (BF_incl_ = 1.204 for the group x time interaction effect).

On average, athletes trained 12.3 ± 4.2 h/week at week 0 and decreased their total training volume during the study to 10.6 ± 5.1 h per week (BF_incl_ = 10.92), time in intensity zone 1 (BF_incl_ = 30.31) and 2 (BF_incl_ = 45.47) but showed no support for an interaction effect between group and measurement time point. Time in intensity zone 3, 4, and 5 and non-exercise activity thermogenesis did not change from the baseline to week 17 in any of the groups. A more detailed description of the activity characteristics can be found in Table S2.

## 4. Discussion

This is the first sports nutrition intervention study on nutrition knowledge and dietary behavior changes in female endurance athletes with symptoms of RED-S, categorized by a LEAF-Q score ≥ 8. We conducted an international multicenter study with a theoretical framework and standardized procedures combining weekly online lectures and individual consultations every other week. The main finding was strong evidence of improved sports nutrition knowledge, but weak evidence of an improvement in dietary behavior after the sixteen weeks of intervention.

The FUEL intervention group showed a 28% improvement in their sports nutrition knowledge during the 16-week intervention period. This is higher than the mean increase of 16% based on the results of 32 studies reviewed by Tam and colleagues, investigating the effectiveness of educational interventions designed to improve nutrition knowledge in athletes [32]. Overall, the included studies in their systematic review used interventions that were primarily delivered in a face-to-face group setting, were short-term (typically < 4 weeks with a total contact time of <300 min), and had a variety of session frequencies (from a single session to yearly sessions) and durations for each session (usually ≤ 1 h). Studies using online content had a lower attrition range, while the knowledge scores increased compared to non-technology-based education protocols. In the present study, the longer duration of the intervention, the higher overall contact time (558 min), and the use of an online approach may in part explain the improvement in nutrition knowledge compared to the findings in the systematic review by Tam et al. [32]. Because tier 3–4 athletes often have significant time challenges due to high training load in combination with either work or studies, online lectures that can be seen whenever is suitable in the athletes’ everyday lives may offer a sustainable means of supporting athlete education [32]. Additionally, as the individual consultations in the present study also were online, athletes could avoid transportation time spent to meet their nutrition counselor, which may improve retention rates, even in non-pandemic times.

The addition of individual athlete-centered consultations to the lectures may also be an advantage for improving nutrition knowledge, since this gives athletes the opportunities to ask questions about the lectures, and discuss individual challenges and how to implement optimized strategies into their everyday lives, thereby stimulating procedural knowledge, not only declarative knowledge [66]. Although education programs are needed within the field of RED-S [25], and are frequently used behavioral strategies to promote dietary behavior change in athletes [67], the association between nutrition knowledge and dietary intake was found to be modest (*r* < 0.44) in a systematic review [68]. Despite a positive relationship, several other factors influence food intake in athletes, including social and economic factors, [22,23] and it is often argued that nutrition knowledge is a necessary but not a sufficient factor for changing behavior [31,66,68], which is also implied by the results of the present study.

While we found strong evidence of improved nutrition knowledge in the intervention group, we found limited evidence of changes in their dietary intake. This discrepancy was supported by the ranking of the statement “There has been agreement between how I have eaten and my theoretical knowledge of sports nutrition”, which was the only one of five subjective rankings with no group x time interaction effect, reflecting the complexity of changing habits, despite improved knowledge [52,66]. When splitting the FUEL intervention group into those with the highest versus the lowest change in sports nutrition knowledge score, and into those with the highest versus the lowest sports nutrition knowledge score post-intervention, we did not find evidence of differences in changes in any dietary outcomes, supporting the abovementioned incomplete association between nutrition knowledge and behavior. It could be argued that athletes’ initial readiness to change could influence the effect of the intervention. Based on The Transtheoretical Model, the majority (56%) of the athletes placed themselves in the *preparation phase*, while 22% placed themselves in the *contemplation phase*, and the remaining 22% placed themselves in the *action phase*. We did not, however, find evidence of any group × time interaction effects for the dietary outcomes comparing these three groups.

Improved sports nutrition knowledge without a corresponding improvement in dietary intake has also been reported in earlier intervention studies of endurance athletes. Three 90 min education sessions with and without a mobile phone application improved nutrition knowledge, but not dietary intake, among 79 male and female endurance athletes aged between 16–20 [69]. Likewise, Dickey and Nolte reported improved nutrition knowledge among seven female rowers after eight individual sports nutrition counseling sessions and eight co-active life coaching sessions, but dietary intake did not improve, and energy availability was still low or reduced for all seven participants after the intervention [70]. Contrarily, in a systematic review by Bentley et al. [67] investigating behavioral strategies used to promote dietary behavior change in athletes, most studies reported changes in athletes’ dietary behavior post-intervention. The participants in the included studies were, however, mostly from ball game sports and were not specifically identified as being at risk of RED-S like the athletes in the present study. In addition, many of the studies in the aforementioned review have been criticized for using few of the available behavior change techniques, and for not reporting which theory grounds are used as the basis for the intervention [67]. Even though we used an evidence-based theoretical framework in the present study, we only found weak evidence of an improvement in dietary intake compared to the control group. We, therefore, speculate whether it may be extra challenging for female endurance athletes with symptoms of RED-S to improve their dietary behavior, for e.g., increase their energy intake, due to their focus on maintaining a lower body mass [16], a limited time to eat because of a busy training schedule or due to suppression of appetite after training [8,9]. Being a non-professional competitive athlete with studies and/or work in addition to a busy training schedule may add extra challenges to behavior change and meeting energy requirements. It could also be speculated as to whether athletes at different ages received and responded differently to the FUEL intervention, as a 35-year-old athlete may have more ingrained habits compared to an 18-year-old athlete. Indeed, a sub-analysis revealed weak evidence of a greater improvement (BF_incl_ =1.575) in carbohydrate intake (g/kg/day) among FUEL athletes ≥ 26 years (4.8 ± 1.1 at week 0 and 5.9 ± 1.3 at week 17) of age (the median) compared to athletes < 26 years of age (4.9 ± 1.0 at week 0 and 5.2 ± 1.3 at week 17). Another possibility for differential receptivity to the intervention could be due to sport modality, because perceived social norms for healthy eating may be more powerful in some sports compared to others; we have previously reported a higher risk of LEA among runners compared to triathletes, cyclists, biathletes, and cross-country skiers [7]. We did not, however, find evidence of differences in change according to sport modality in the outcomes relevant to this analysis.

In line with previous research on female endurance athletes with symptoms of RED-S [16,17,18,19], participants in the present study had a carbohydrate intake below the recommended minimum requirement of 6 g/kg/day [61] and a dietary fiber intake above the recommended 25–35 g/day [71] at both time points. A low intake of carbohydrate-rich foods and a high intake of dietary fibers have been associated with symptoms of RED-S in female endurance athletes [16], and were therefore important topics in the nutrition lectures and the individual consultations in the FUEL intervention. Although 74% of the athletes in the FUEL intervention group increased their carbohydrate intake from the baseline to post-intervention (12% increase in g carbohydrate/day and 15% increase in g carbohydrate/kg/day), our results suggest that this group of athletes only met the carbohydrate recommendations a few days a week, even after the nutrition intervention. Since the athletes in the FUEL intervention group decreased their training volume from the baseline to post-intervention and increased their daily energy intake by 5% it would have been interesting to measure the potential changes in energy availability. Unfortunately, assessment of the lean body mass was not possible in the present study due to the COVID-19 restrictions prohibiting the athletes from traveling to the lab to participate in dual-energy X-ray absorptiometry scans. When comparing those who had the greatest reduction in training volume with those with a minor reduction in training volume in the intervention group (median split), we did not find evidence of differences in changes in energy intake or carbohydrate intake from the baseline to post-intervention. Łagowska et al. (2014) reported improved energy intake (+234 kcal/day) and energy availability (+7.5 kcal/kg FFM/day) among 45 female athletes with menstrual dysfunction after a three-month intervention, where athletes had been informed of nutritional mistakes and were given an individual diet [40]. After extending the study to nine months, the researchers reported a more profound increase in energy intake and energy availability [72]. That study is, however, limited by the lack of a control group and does not report which theory and behavior change method(s) they have used as a basis for their intervention, but can nevertheless indicate that a longer intervention duration may be needed for female athletes to change their dietary behavior. Among non-athletes, it has been suggested that long-term education programs (>5 months) have the best efficacy on improving nutrition behaviors [73]. It is possible that a longer intervention in the FUEL study could have enabled further motivation for change and supported more athletes during the *action phase*.

### 4.1. Strengths and Limitations

A novel aspect of our study, which also represents an underlying strength, is the combination of online lectures and individual consultations, which were athlete-centered and aimed at inducing behavioral change. We used behavior change theories and approaches to promote standardization in the intervention between the countries and sports nutritionists, which were all highly experienced in working with athletes with RED-S. However, since the consultations were not recorded, we are not able to evaluate to what degree the attended theories and approaches were used. Recording the consultations, on the other hand, may have prevented the athletes from being open about their feelings and behaviors.

In contrast to other intervention studies involving female athletes with symptoms of RED-S [74], it is a strength that the present study included a control group. It would be interesting to include additional groups (a group receiving FUEL lectures only and a group receiving FUEL consultations only), for the purpose of identifying the active components of different outcomes. Another strength was the exclusion of athletes with a risk of disordered eating, those using hormonal contraceptives and athletes with previously diagnosed menstrual dysfunctions not related to RED-S, whereby the likelihood of including false positive RED-S cases was decreased.

Although we recruited athletes from four different countries, the strict inclusion criteria resulted in a relatively low number of participants. As laboratory assessments were canceled, athletes’ only motivation for participating in the study may have been the intervention itself. This may explain the particularly low number of controls. Although controls were offered during the FUEL intervention after the 16-week control period, this may have been too long a period to keep them in the study.

Educational studies have been criticized for using a wide range of knowledge assessment tools with limited validation, which makes it difficult to compare results across studies [32,75]. In the present study, we developed twenty statements specifically relevant for female endurance athletes with a risk of RED-S that were suitable for a short telephone interview, so that participants were not able to find the answers in books or on the internet which they could have done with an online distributed questionnaire. Our knowledge tool is an abbreviated version of what has been used in previous studies [32,69,75]. Consequently, scores may not be fully representative of sports nutrition knowledge and direct comparisons with other studies are not possible. Despite these mentioned limitations, it was the development from the baseline to post-intervention compared to the development from the baseline to post-test for the control condition that was in focus, thereby making the applied method suitable for the present study.

Finally, it is a limitation that the data assessment was conducted at different phases of the athletic season for the intervention group and the control group. This reduces the comparability between the two groups considering the periodization of training, and energy intake and carbohydrate intake. However, an observational study reported persistent LEA throughout the season among triathletes [76], suggesting that endurance athletes do not necessarily periodize their dietary intake. In addition, data from the present study shows the same reduction in training volume in both groups. Nevertheless, it is important to acknowledge the numerous existing interferences with athletes’ nutritional behaviors, beyond the athletic season, including but not limited to interpersonal factors, influences from social media and marketing, the weather, and life situations that may affect their mood, which are outside the influence of this study [31].

### 4.2. Future Directions

Given the complexity of behavior change, future studies should start early and aim for primary prevention, thereby including junior athletes. In addition, due to increased knowledge of RED-S also among male athletes [2], future studies should also include male participants. Further, coaches from endurance sports who dictate the energy demands of training and are in a prime position to observe changes in athletes’ health and performance, are not well enough educated about RED-S [77]. Cultural revolutions and changes in social norms are needed [25], and therefore, future studies should not only include individual athletes, but also their coaches, health professionals, entire teams, clubs, and sports organizations. Future studies are encouraged to use a systematic needs assessment in the planning phase using a framework (e.g., using worksheets from the behavior change wheel [78]) and user engagement.

### 4.3. Practical Implications

The recommended treatment for improving energy intake seems straightforward at first glance but is complex in practice. Providing information about optimal sports nutrition strategies, with the aim of correcting dietary behavior, maybe commonplace when RED-S is suspected, at least among elite athletes with access to a professional sports nutrition team. Practitioners should, however, be aware that improving sports nutrition knowledge is not necessarily sufficient for behavior change in female endurance athletes with a risk of RED-S [33], which is also indicated by the results of the present study. Even with the combination of educational lectures, specifically designed for the target group, and individual consultations including behavior change techniques such as goal setting and social support, the present study implies that additional initiatives should be implemented. Of importance, however, the recommended treatment targets for LEA should be gradual and accomplished over several months [35], and therefore, an intervention period of 16 weeks may be insufficient.

## 5. Conclusions

Our results provide strong evidence that the FUEL intervention improved sports nutrition knowledge among female endurance athletes with symptoms of RED-S. Although improvements in dietary behavior were only modest, the FUEL intervention shows promise as a foundation for behavior change in female endurance athletes at risk of REDs.

## Figures and Tables

**Table 1:** Structure of the FUEL consultations inspired by the Four Habits Model [53].

Preparation	Beginning	Work Part	Conclusion	Reflection
Athlete takes notes and summarizes to herself what is important to bring up during the lecture The counselor prepares conversation based on template for conversations and previous conversations as well as online lectures viewed by the athlete	Establish contact (specifically for the first consultation: establish trust, identify the motivation for change, inform about further course progression) Engage Focus Set point: What does the athlete experience as important (today, the next weeks and in the future)?	Explore the athlete’s perspective (thoughts, feelings) of their personal situation related to RED-S, e.g., potential symptoms, dietary restrictions Wish and intention to make changes Goal, action plans, and approaches Support mastery, loyalty, and trust in one’s own ability to conduct change/mastery (including exploring previous experiences) Inform	Explore the athlete’s experience of the consultation Ask for additional questions Summarize and conclude	The athlete writes personal notes The counselor writes a journal
← Maintain alliance ∙ Express empathy ∙ Support autonomy ∙ Support mastery ∙ Give information →				

**Table 2:** Participant characteristics are divided by intervention (FUEL) and control (CON) groups.

	FUEL (*n* = 31)	CON (*n* = 15)
Age (years)	24.1 ± 4.7	25.3 ± 4.8
Height (cm)	169.3 ± 6.2	171.2 ± 7.1
Body weight (kg)	59.5 ± 7.0	59.3 ± 5.0
BMI (kg/m^2^)	20.8 ± 2.1	20.3 ± 1.7
Training volume (h/month)	46.3 ± 16.7	48.6 ± 17.7
Full-time athlete (%)	16.1	20.0
Occupation ^1^		
Full-time job (%)	45.2	33.3
Part-time job (%)	0.0	0.0
Studying (%)	38.7	40.0
Other (%)	0.0	6.6
Level of competition		
Club (%)	64.5	86.7
National team (%)	19.4	6.7
Professional (%)	9.7	6.7
Other (%)	6.5	0.0
Level of education		
Primary school (%)	0.0	0.0
Secondary school (%)	25.8	46.7
University/college < 4 years (%)	48.4	20.0
University/college ≥ 4 years (%)	25.8	33.3

Continuous data are presented as mean ± SD and categorical data as a percentage. ^1^ Athletes who responded that they were not full-time athletes were asked about their occupation beyond their endurance sport. Abbreviations: BMI: body mass index; CON: control group; FUEL: the FUEL intervention group.

**Table 3:** Self-perceived sports nutrition knowledge divided by intervention (FUEL) and control (CON) group.

	FUEL			CON			
Statements Rated on a Scale from 1 to 10 (1 = Totally Disagree, 10 = Fully Agree)	Week 0	Week 17	Within Group Difference	Week 0	Week 17	Within Group Difference	BF_incl_
I have great knowledge in the field of sports nutrition	5.8 ± 1.9	8.1 ± 1.5	2.2 ± 1.6	7.0 ± 1.5	6.7 ± 1.9	−0.3 ± 1.6	592.02
I have followed all sports nutrition recommendations I can	5.3 ± 1.8	7.6 ± 1.7	2.2 ± 2.0	5.8 ± 1.9	5.3 ± 1.8	−0.4 ± 2.2	68.88
There has been agreement between how I have eaten and my theoretical knowledge of sports nutrition	6.4 ± 1.6	7.8 ± 1.4	1.4 ± 1.9	4.5 ± 1.4	4.8 ± 2.2	0.3 ± 2.8	0.94
I have confidence in my nutrition routines	6.0 ± 1.6	7.8 ± 1.4	1.8 ± 2.2	5.8 ± 2.0	5.8 ± 2.1	0.1 ± 2.8	9.38
I have known where I should gather research-based information about sports nutrition	5.3 ± 2.9	8.7 ± 1.6	3.5 ± 2.8	6.0 ± 2.9	6.8 ± 2.7	0.8 ± 3.1	8.70

Data are presented as mean ± SD. For the FUEL intervention group, post data for self-perceived sports nutrition knowledge were missing for *n* = 1 participants, while post data were missing for *n* = 3 participants in the control group. Abbreviations: BF_incl_ = Bayes factor for inclusion of group × time interaction; CON: control group; FUEL: the FUEL intervention group.

**Table 4:** Dietary characteristics for the intervention (FUEL) and control (CON) group.

	FUEL			CON			BF_incl_
Dietary Intake	Week 0	Week 17	within Group Difference	Week 0	Week 17	within Group Difference	
Energy intake (kcal/day)	2588 ± 528	2726 ± 547	138 ± 453	2455 ± 482	2300 ± 449	−155 ± 396	1.03
Carbohydrates (g/day) Carbohydrates (g/kg/day) Carbohydrates (E%)	290 ± 68	326 ± 88	36 ± 74	285 ± 65	280 ± 74	−6 ± 62	1.09
	4.8 ± 1.0	5.5 ± 1.4	0.6 ± 1.3	4.8 ± 1.0	4.7 ± 1.2	−0.1 ± 1.0	1.04
	47 ± 8	50 ± 8	2.7 ± 9.4	49 ± 5	51 ± 6	2.2 ± 5.4	0.38
Dietary fibers (g/day)	37.5 ± 12.5	36.7 ± 12.7	−1 ± 10	36 ± 9	37 ± 15	1 ± 12	0.37
Dietary fibers (g/1000 kcal)	14.4 ± 3.8	13.3 ± 3.1	−1.1 ± 3.2	14.9 ± 3.6	15.9 ± 5.2	1.0 ± 4.7	0.88
Protein (g/day)	107 ± 30	115 ± 31	8 ± 22	95 ± 19	88 ± 24	−7 ± 17	1.06
Protein (g/kg/day)	1.8 ± 0.5	1.9 ± 0.5	0.1 ± 0.4	1.6 ± 0.3	1.5 ± 0.4	−0.1 ± 0.3	1.12
Protein (E%)	17 ± 4	18 ± 4	3 ± 3	17 ± 4	16 ± 4	−1 ± 2	0.42
Fat (g/day)	106 ± 35	96 ± 26	−10 ± 34	97 ± 24	88 ± 23	−9 ± 23	0.36
Fat (g/kg/day)	1.8 ± 0.6	1.6 ± 0.4	−0.2 ± 0.6	1.6 ± 0.3	1.4 ± 0.4	−0.2 ± 0.4	0.38
Fat (E%)	37 ± 9	32 ± 6	−5 ± 9	35 ± 5	34 ± 7	−1 ± 7	1.43

Data are presented as mean ± SD. A total of *n* = 6 athletes in the control group had missing or incomplete dietary records. Analyses were therefore available for *n* = 31 athletes in the intervention group and *n* = 9 athletes in the control group. Abbreviations: BF_incl_ = Bayes factor for inclusion of group × time interaction; CON: control group; FUEL: the FUEL intervention group.

## Data Availability

The raw data supporting the conclusions of this article will be made available by the authors, without undue reservation.

## Supplementary Materials

The following supporting information can be downloaded at: https://www.mdpi.com/article/10.3390/nu15051082/s1, File S1: Scoring key for sports nutrition-related behavior, Table S1: Sport nutrition knowledge; Table S2: Training and activity characteristics.

## Abbreviations

ANOVA	Analysis of variance
BF	Bayes factor
BF_incl_	Bayes factor for inclusion of group x time interaction
BMI	Body mass index
CON	Control group
EDE-Q	Eating Disorder Examination Questionnaire
E%	Energy percentage
FUEL	Food and nUtrition for Endurance athletes—a Learning program
g	gram
HC	Hormonal contraceptives
kcal	Kilocalorie
kg	Kilogram
LEA	Low energy availability
LEAF-Q	Low Energy Availability in Females Questionnaire
RED-S	Relative Energy Deficiency in Sport
SD	Standard deviation
TSD	Services for Sensitive Data
