# Supplementary material for: Effects of a 16-Week Digital Intervention on Sports Nutrition Knowledge and Behavior in Female Endurance Athletes with Risk of Relative Energy Deficiency in Sport (REDs)
Source: Nutrients. 2023 Feb 21;15(5):1082. doi: 10.3390/nu15051082 (PMC10005555; doi:10.3390/nu15051082)
Supplement: Supplementary file 1 [file nutrients-15-01082-s001.zip › S1. Scoring key sports nutrition related behavior.pdf]

Supplementary: Scoring key for sports nutrition related behavior

1) How often have you consumed carbohydrate containing drink/food during training sessions lasting more than 1 hour?
Never = 0
Sometimes = 1
Often = 2
Always = 3

2) Have you eaten breakfast before an early morning session?
Never = 0
Sometimes = 1
Often = 2
Always = 3

3) How often have you eaten a snack or meal right after a training session (within 30 min)?
Never = 0
Sometimes = 1
Often = 2
Always = 3

4) How often have you eaten carbohydrate-rich foods (for example bread, rice, pasta, potatoes, or cereal/oats)?
<1 time per day = 0
1 time per day = 1
2-3 times per day = 2
4-5 times per day = 3
≥ 6 times per day = 4

5) Have you practiced nutrition strategies before an important competition? (e.g. do you test your pre-race meal and mid-race fluids)?
Never = 0
Sometimes = 1
Often = 2
Always = 3
Not relevant = missing

6) Have you had routines that allows you to increase your carbohydrate stores before long-lasting competitions (> 90 min)?
Never = 0
Sometimes = 1
Often = 2
Always = 3
Not relevant = missing

7) Have you changed your energy intake when you have increased your training load?
When I have trained more, I have eaten less = 0

I have eaten the same amounts regardless of how much I have trained = 0
When I have trained more, I have eaten more = 3

Before an important training session or competition, I have tried to…
8) increase my carbohydrate intake = 3
9) limit my fiber intake = 2
10) increase my fluid intake = 3
