# Supplementary material for: Effects of a 16-Week Digital Intervention on Sports Nutrition Knowledge and Behavior in Female Endurance Athletes with Risk of Relative Energy Deficiency in Sport (REDs)
Source: Nutrients. 2023 Feb 21;15(5):1082. doi: 10.3390/nu15051082 (PMC10005555; doi:10.3390/nu15051082)
Supplement: Supplementary file 1 [file nutrients-15-01082-s001.zip › Table S1. Nutrition knowledge.pdf]

Table S1. Correct answers from the telephone interview on sports nutrition knowledge

	FUEL		CON	
**Statements (correct answer)**	**Week 0**	**Week 17**	**Week 0**	**Week 17**
I should eat the same types of food and amounts every day (false)	83%	100%	100%	100%
My vitamin- and mineral needs are so high that it is hard to fulfill through ordinary food (false)	72%	93%	70%	80%
The absence of menstruation for longer or short periods is due to a high training load per se (false)	31%	59%	20%	20%
An athlete who is amenorrheic have the potential to regain her period through eating more food (true)	83%	100%	50%	80%
It is smart for me to train fasted (false)	76%	97%	80%	90%
Protein supplements contribute to a better protein synthesis than protein from ordinary food (false)	93%	100%	90%	80%
Athletes who are amenorrheic often have a lower resting metabolic rate than those who are eumenorrheic (true)	31%	83%	20%	30%
I should avoid eating saturated fat (false)	83%	86%	30%	10%
I should avoid eating food and drink with added sugar like cakes and other treats (false)	83%	100%	20%	20%
Endurance training does not increase my daily need for protein (false)	83%	90%	90%	90%
I should choose low fat foods in order to maintain a low weight (false)	93%	100%	90%	100%
I should supplement with vitamins and minerals to make sure I get enough (false)	41%	79%	80%	60%
I should consume carbohydrates on training sessions and competitions lasting >1,5 hours (true)	100%	100%	100%	100%
For me there is no need to eat a recovery meal (false)	97%	100%	90%	100%
It is difficult to get enough protein from ordinary food (false)	86%	100%	60%	80%
Whole wheat products are what I should choose as my main carbohydrate source (false)	35%	83%	20%	10%
I need more iron than other female athletes who are not endurance athletes (true)	76%	79%	60%	70%
A high dietary fiber content may be the reason for an insufficient energy intake (true)	41%	90%	40%	50%
As a female endurance athlete, I should maintain the same body weight throughout the year (false)	86%	97%	80%	80%
Fruit and vegetables are sufficient snacking meals for me (false)	48%	97%[c]	20%	10%
**Total number of correct answers**	14.3 ± 2.6	18.3 ± 1.5	12.1 ± 2.6	12.6 ± 2.2

For the FUEL intervention group, post data for the telephone interview were missing for *n*=2 participants. For the control group, pre data for the telephone interview were missing for *n*=2 participants and pre and post data were missing for *n*=3 participants. Abbreviations: FUEL: the FUEL intervention group, CON: control group.
