# Supplementary material for: Effects of a 16-Week Digital Intervention on Sports Nutrition Knowledge and Behavior in Female Endurance Athletes with Risk of Relative Energy Deficiency in Sport (REDs)
Source: Nutrients. 2023 Feb 21;15(5):1082. doi: 10.3390/nu15051082 (PMC10005555; doi:10.3390/nu15051082)
Supplement: Supplementary file 1 [file nutrients-15-01082-s001.zip › Table S2. Training and activity characteristics.pdf]

Table S2. Physical activity characteristics for the FUEL intervention group and the control group

	FUEL		CON		BF_{incl}
	**Week 0**	**Week 17**	**Week 0**	**Week 17**	
**Physical activity**					
Total training volume (h/week)	12.1 ± 3.7	10.8 ± 3.9	12.4 ± 4.6	10.4 ± 6.2	0.408
I1 (min/week)	267 ± 204	148 ± 103	242 ± 152	144 ± 60	0.386
I2 (min/week)	208 ± 113	148 ± 103	195 ± 73	144 ± 60	0.383
I3 (min/week)	71 ± 45	47 ± 35	52 ± 42	50 ± 27	0.632
I4 (min/week)	41 ± 40	33 ± 27	21 ± 16	17 ± 23	0.380
I5 (min/week)	13 ± 25	9 ± 18	6 ± 9	5 ± 8	0.370
NEAT (kcal/day)	241 ± 154	236 ± 135	343 ± 180	244 ± 105	2.085

Abbreviations: BF_{incl} = Bayes factor for incluision ofgroup * time interaction, CON: control group, FUEL: FUEL intervention group, NEAT: Non Exercise Activity Thermogenisis, NS: No support for the group x time interaction effect being the best model.
